# Collaborative Frontiers in Pediatric Neuro-Oncology: Establishing an International Tumor Board for Enhanced Care and Global Impact

**DOI:** 10.21203/rs.3.rs-5799306/v1

**Published:** 2025-04-21

**Authors:** Margaret Shatara, Nicole M. Brossier, Andrew Cluster, Ali Y. Mian, Sonika Dahiya, Amy E. Armstrong, Angela C. Hirbe, David H. Gutmann, Kenneth Aldape, Mohamed S Abdelbaki

**Affiliations:** Children’s Minnesota; Washington University In St Louis: Washington University in St Louis; Washington University In St Louis: Washington University in St Louis; Washington University In St Louis: Washington University in St Louis; Washington University In St Louis: Washington University in St Louis; Washington University In St Louis: Washington University in St Louis; Washington University In St Louis: Washington University in St Louis; Washington University In St Louis: Washington University in St Louis; National Cancer Institute; Washington University In St Louis: Washington University in St Louis

**Keywords:** Pediatric Neuro-Oncology, CNS Tumors, Internationmal Collaboration, Telemedicine, Global Health Disparities

## Abstract

**Background:**

Central nervous system tumors are the leading cause of cancer-related mortality in children, with significant disparities in diagnostic and treatment capabilities between low- and middle-income countries and high-income countries. This study outlines the establishment of an international neuro-oncology tumor board to address these gaps.

**Methods:**

The tumor board was initiated in January 2021 through a partnership between Washington University in St. Louis, USA, and nine institutions, ultimately expanding to 39 institutions across 25 countries. Monthly virtual meetings facilitated multidisciplinary case reviews offering diagnostic and management recommendations. A retrospective analysis of 29 sessions over three years was conducted, and a cross-sectional web-based survey among participants assessed their experiences and satisfaction.

**Results:**

From January 2021 to December 2023, 101 cases were reviewed. The most diagnoses were low-grade gliomas (23.4%) and neurofibromatosis type 1 and 2 (32.7%). Newly diagnosed cases comprised 51%, while 40% involved recurrent or progressive disease, and 9% were inquiries during ongoing therapy. Recommendations predominantly addressed therapeutic strategies (60.7%). Advanced diagnostics, such as methylation profiling, refined diagnoses in several cases. The survey, with a 35% response rate, showed high satisfaction, with 91% finding the meetings educational. Barriers included time constraints (71%) and conflicting commitments (27%).

**Conclusion:**

This initiative, to our knowledge, represents the largest international pediatric neuro-oncology tumor board. Multidisciplinary discussions improved diagnostic precision, informed therapeutic decision-making and facilitated educational exchange. Participants reported positive impacts on professional development and alignment with institutional needs. Despite challenges, this study highlights telemedicine’s potential to bridge resource disparities and improve the outcomes globally.

## Introduction

Brain and spinal cord tumors are the second most common cancer in children after leukemia, accounting for approximately 25% of all childhood cancers. Annually, over 4,000 new cases are diagnosed globally ^1^. Outcomes in low- and middle-income countries (LMICs) demonstrate significant disparities when compared to high-income countries, highlighting the critical need for collective efforts to participate in improving the care in these countries^2,3^. The broad spectrum of clinical, pathological, and biological subtypes presents significant challenges to research and clinical trials, underscoring the importance of international collaboration ^4,5^. The inherent complexity of pediatric central nervous system (CNS) tumors not only complicates therapeutic strategies but also has profoundly impacts long-term health outcomes, necessitating a globally coordinated approach to enhance understanding and improve care in this domain ^6^. Moreover, survivors of CNS tumors often face chronic health conditions and neurological impairments, necessitating a multidisciplinary approach to treatment ^6,7^. The lack of coordinated care and limited access to specialized facilities in LMICs pose substantial barriers to optimal management in these children ^8–10^.

In response to these challenges, international collaborations have emerged as vital conduits for improving pediatric neuro-oncology care. For example, the World Health Organization’s Global Initiative for Childhood Cancer (GICC) was established to improve survival rates for pediatric cancer patients to at least 60% by 2030, identifying low-grade glioma as one of its six index cancers and demonstrating the need for targeted interventions ^11^.

Collaborative initiatives, such as twinning programs and cross-border partnerships, have consistently demonstrated improved pediatric neuro-oncology care in LMICs. These programs, often leveraging telemedicine, have enhanced diagnostic capabilities, facilitated access to advanced therapies, and significantly improved clinical outcomes while fostering local capacity building ^12–16^. For example, the long-standing partnership between the King Hussein Cancer Center (KHCC) in Jordan and the Hospital for Sick Children (SickKids) in Toronto ^14^, as well as the Cross-Border Neuro-Oncology Program between Rady Children’s Hospital in San Diego and Hospital General in Tijuana ^16^, highlighted the role of telemedicine in refining treatment plans and increasing survival rates.

In contrast to these bilateral efforts, our international pediatric neuro-oncology tumor board represents a novel approach by initiating a multi-country collaboration from its inception. The board integrated institutions from four countries at its launch and subsequently expanded to 25 countries over three years, establishing a global framework for multidisciplinary knowledge exchange and capacity building.

In this manuscript, we present the establishment of an international, multi-disciplinary pediatric neuro-oncology tumor board, which was initiated in January 2021. Our report encompasses a thorough examination of the board’s three-year experience, detailing the challenges encountered, strategies implemented, and prospective avenues for advancing care for children afflicted with CNS tumors across varied healthcare landscapes. By examining the impact of collaborative efforts, we seek to contribute to the ongoing discourse on improving outcomes for pediatric neuro-oncology patients globally.

## Methods

The international pediatric neuro-oncology tumor board was initiated as a pilot project in January 2021, fostering collaboration between Washington University School of Medicine in St. Louis, Missouri, USA, and nine international sites across four countries: Egypt, Saudi Arabia, Lebanon, and Jordan. This initial collaboration subsequently expanded to encompass 39 institutions from 25 countries by December 2023. Our team comprises experts in Pediatric Oncology, Pediatric Neurosurgery, Adult and Pediatric Neurology, Neuropathology, Neuroradiology, and Radiation Oncology, representing a wealth of knowledge from all participating institutions.

In September 2021, we integrated a quarterly Neurofibromatosis (NF) meetings into our initiative, leveraging the expertise of specialists from the Washington University NF Center (https://nfcenter.wustl.edu).

Meetings were held monthly *via* “Zoom” videoconferencing platform, and typically lasting between 60–90 minutes. Cases were submitted prior to the meeting for thorough review by our multidisciplinary team. Moreover, our collaborative efforts extended beyond the confines of traditional tumor board discussions. For select cases, we offered additional services, including specialized radiology and pathology reviews. Furthermore, we facilitated access to advanced methylation profiling services through The National Institutes of Health (NIH).

We conducted a retrospective analysis of the videoconference sessions held between January 2021 and December 2023. Detailed minutes from each session were retrospectively reviewed, and pertinent data were extracted from the accompanying PowerPoint presentations. This dataset encompassed various aspects including demographic information, clinical findings, radiographic images, pathological assessments, primary diagnostic outcomes, treatment modalities pursued, recommendations put forth, and subsequent follow-up details where applicable. Additionally, we systematically examined and summarized the prevalent reasons for consultation during these sessions.

In September 2023, recognizing the growing participation in the program, we conducted a web-based survey to assess participant experiences and satisfaction, aiming to optimize this international collaboration. The cross-sectional survey was distributed via a web-based platform to participating healthcare professionals, ensuring convenient access for participants from diverse geographical locations. The survey, conducted via RedCap and approved by the institutional review board, consisted of two parts: one focused on the monthly multidisciplinary international tumor boards and the other tailored to the quarterly NF tumor boards. Participants provided feedback and insights separately for each aspect of the tumor board sessions.

We report the descriptive analysis of the demographic characteristics, primary diagnoses, treatment strategies, recommendations, and consultation patterns observed during the tumor board sessions, aiming to provide valuable insights into the clinical characteristics, management strategies, and recommendations discussed within our international tumor board. We also present the results of the cross-sectional survey.

## Results

### Case Presentations and Demographics

**Figure 1:**
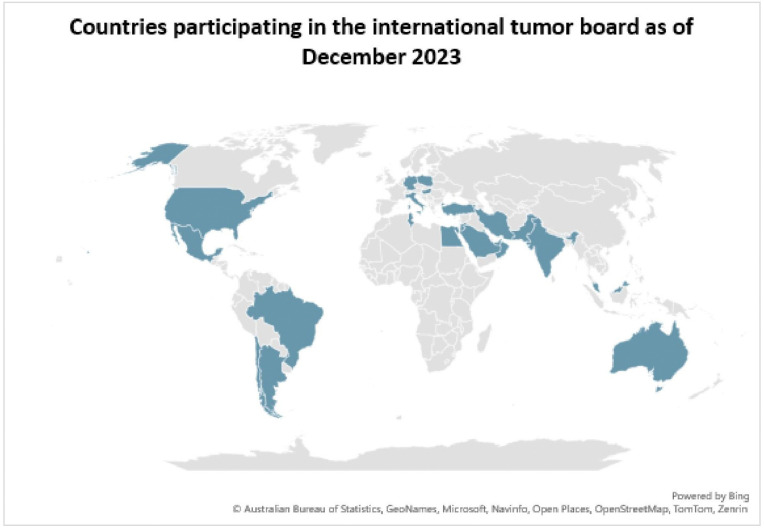
Countries involved in the international pediatric neuro-oncology tumor board as of December 2023.

Between January 2021 and December 2023, 29 virtual meetings were conducted to review complex cases of pediatric brain and spine tumors. These sessions aimed to address challenging diagnostic and treatment scenarios, with a focus on collaborative decision-making. A total of 101 cases were presented, with the number of cases per meeting ranging from 1 to 8. The participating institutions grew steadily over this period, reflecting increased engagement from across 25 countries (Figure 1). Supplementary Table 1 outlines the distribution of cases by country.

**Figure 2:**
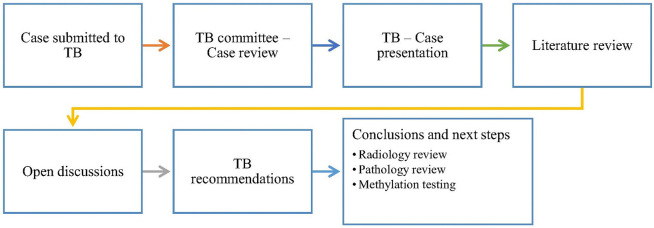
Flow chart of virtual multi-disciplinary tumor board meetings. TB: tumor board.

Complex cases of pediatric brain and spine tumors were presented and discussed at each meeting. These cases may involve newly-diagnosed patients, recurrent tumors, or challenging treatment decisions. During the sessions, the primary neuro-oncologist/ oncologist presented the case, which was then discussed in depth, considering relevant literature and treatment options. Tumor board members review the clinical information, radiological imaging, pathology reports, and other relevant data to collectively recommend the best course of action for each patient. Our discussions led to comprehensive recommendations and documented strategies for further action, ensuring all participants were informed. Patient privacy was rigorously maintained through de-identification protocols (Figure 2). Cases were submitted ahead of the meeting to allow our multidisciplinary team ample time for comprehensive review. The tumor board leader conducted a meticulous evaluation of all cases, ensuring that all images, videos, and reports were thoroughly de-identified, thereby safeguarding patient privacy during Zoom sessions.

Of the 101 cases discussed, 97 (96%) were presented to address specific clinical inquiries, while 4 cases (4%) were shared for their educational value. The median age of patients at the time of discussion was 9 years (range: 2 months to 31 years). Newly diagnosed cases comprised 51% of the total, 40% involved recurrent or progressive disease, and 9% were inquiries made during ongoing therapy.

**Figure 3:**
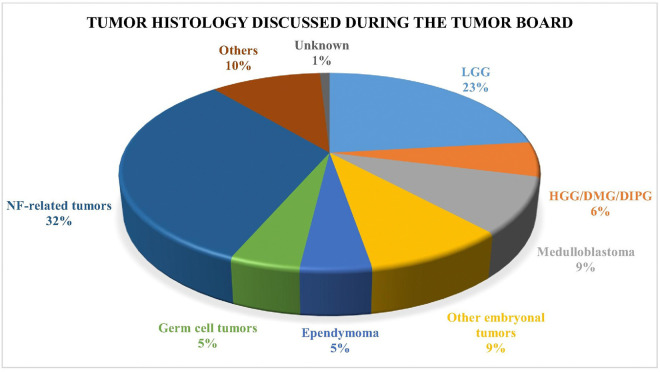
Distribution of different pathologic diagnoses discussed. LGG: low-grade gliomas. HGG: high-grade gliomas. DMG: diffuse midline gliomas. DIPG: diffuse intrinsic pontine glioma. NF: neurofibromatosis.

Low-grade gliomas comprised 23.4% of cases, while NF-related tumors accounted for 32.7% (Figure 3). The NF-related cases included both NF1- and NF2-associated tumors, encompassing optic pathway gliomas, low-grade gliomas, neurofibromas, and schwannomas.

### Therapeutic Approaches

Chemotherapy was the most frequently employed treatment (37.6%), followed by observation (15.4%) and radiotherapy (13.7%). Some cases involved discussion regarding targeted therapy or surgical resection, and the treatment data was unavailable for 27.3% of the cases. Table 1 summarized the clinical characteristics of cases discussed.

### Reason for consult

**Figure 4:**
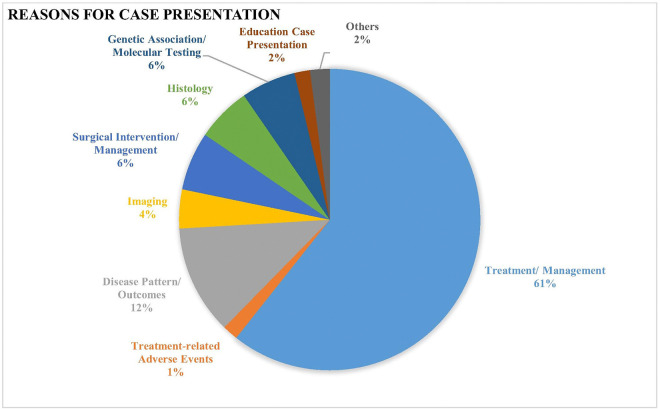
Reasons for case presentation.

The analysis of case presentations (n= 235) revealed that the predominant category (Figure 4), accounting for 60.7% of cases, pertained to treatment and management considerations. highlighting the tumor board’s central role on optimizing therapeutic strategies and ensuring effective patient care in children with CNS tumors. Additionally, 11.7% of presentations focused on disease patterns and clinical outcomes, reflecting the importance of understanding the progression and prognosis of various CNS tumors. Surgical intervention and management were the focal point in 6.28% of cases. Imaging modalities were discussed in 4.2% of cases, and histopathological analysis and molecular/genetic testing each accounted for 5.9% of cases, highlighting the importance of precision diagnostic and prognostic techniques. Treatment-related adverse events and educational case discussions each represented 1.7%. Finally, 2.1% of cases fell under other categories.

### Methylation Testing

In a cohort of seven pediatric cases that underwent methylation profiling, a spectrum of CNS neoplasms was initially diagnosed with distinct clinical presentations and imaging characteristics. Methylation testing provided crucial insights, leading to refined integrated diagnoses for four of these cases. For example, a low-grade glioma with focally elevated Ki-67 was classified with high confidence as a pilocytic astrocytoma (WHO Grade 1), and a pineoblastoma was defined as a pineal parenchymal tumor of intermediate differentiation (WHO Grade 3). The time from initial case discussion to methylation testing discussion ranged from 3 to 7 months, with a median of 4 months. Table 2 summarizes the clinical cases that completed methylation profiling and corresponding results.

The implementation of methylation profiling was initiated to enhance diagnostic accuracy for challenging CNS tumors where traditional methods were inconclusive or ambiguous. Providers from participating institutions utilized this resource to obtain more definitive classifications, which proved instrumental in guiding clinical decision-making. Notably, the testing itself incurred no direct cost to the sending institutions, though they covered the cost of shipping samples to the testing facility. Providers reported that the insights gained from methylation testing were highly valuable in informing treatment plans, particularly for the four cases with refined diagnoses. This underscores the vital role our tumor board played in providing advanced molecular diagnostics as a valuable service to support diagnosis and management of pediatric brain tumors in LMICs.

### Number of cases by year and number of healthcare providers participating

Over the three-year period, there has been a consistent increase in the number of cases presented during our international tumor board meetings, with a noticeable upward trend in both the total number of cases and NF-specific cases. Additionally, there has been a significant increase in the number of healthcare professionals engaging in tumor board discussions. Supplementary Figure 1 illustrates the distribution of cases and participants by year.

### Cross-sectional Survey Results

The survey was completed by 54 respondents from 20 countries, with the majority identifying as pediatric hematology-oncologists (60%) and 10% as neuro-oncologists. Ninety-eight percent of participants attended at least one session over two years, and 72% were actively involved in the quarterly NF meetings. Most participants learned about the tumor board through direct invitation (53%) or via a colleague or mentor (40%), with the remainder through events or participation in studies or projects (7%). Sixty percent attended more than five meetings, while 29% participated in over ten meetings.

The primary barriers to attendance were time constraints (55%) and conflicting professional or personal commitments (28%). A smaller percentage of participants (5%) cited technical issues or lack of awareness as obstacles, with language being a barrier for just one individual. Additionally, 5% of respondents mentioned other reasons for their attendance difficulties, with only two participants specifically noting that time zone differences hindered their ability to attend (Supplementary Figure 2).

**Figure 5:**
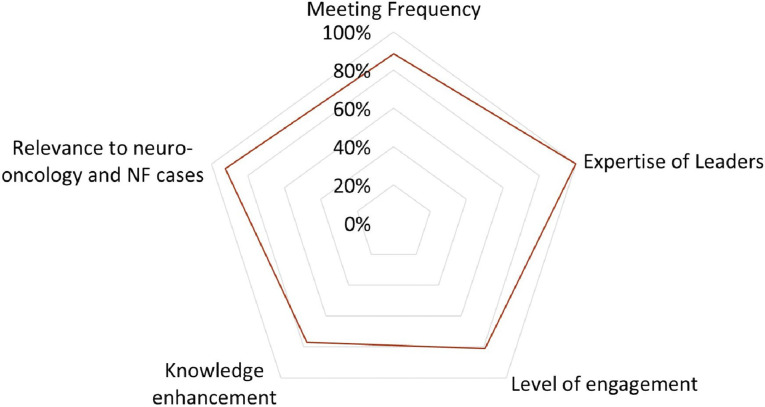
Factors Influencing Tumor Board Participation

Ninety-one percent of respondents noted that the meetings provided valuable educational insights, particularly for challenging pediatric neuro-oncology cases, and 95% recognized the meetings’ positive impact on professional development. Consensus recommendations from the tumor board were perceived as aligning with institutional guidelines (71%), being clear and comprehensible (93%), and contributing to improved outcomes (93%). Factors such as meeting frequency, leader expertise, knowledge enhancement, and engagement level positively influenced participants’ willingness to attend (Figure 5).

## Discussion

The lack of multidisciplinary care poses a significant challenge in managing pediatric CNS tumors in LMICs. The absence of specialized centers, limited infrastructures, delayed diagnosis, restricted access to treatment modalities, and treatment abandonment among factors contributing to suboptimal care and poor outcomes ^9,10^. Several strategies, including international collaborations focused on capacity-building, education, and research, have been employed to address these issues ^10,18,19^.

Twinning programs have emerged as a powerful model for achieving sustained improvements in childhood cancer care in developing countries ^12,20–22^. For instance, telemedicine initiatives, such as the Jordanian-Canadian collaboration, have utilized cost-effective teleconferencing tools to facilitate multidisciplinary case discussions, significantly improving clinical decision-making and patient outcomes ^13,14,15,23^. This program has enhanced local medical expertise, introduced evidence-based treatment strategies, and provided continuous training through knowledge transfer. Similar successes have been observed in other twinning efforts, such as those between SickKids and several hospitals in Pakistan, including Aga Khan University Hospital (AKUH) and Indus Children Cancer Hospital (ICCH), where telemedicine tumor boards have improved diagnostic accuracy and treatment strategies, leading to increased case volume and better management practices.The meetings resulted in a notable decrease in the percentage of plan modifications over time (from 36% in the initial 3.5 years to 16% in the subsequent 3 years), reflecting improved local diagnostic and treatment capabilities ^24,25^.

The Cross-Border Neuro-Oncology Program serves another example of cross-border twinning program collaboration that facilitated significant improvements in clinical outcomes, as evidenced by a dramatic increase in the 5-year overall survival rate, which were attributed to better surgical outcomes, underscoring the importance of skilled neurosurgeons and the benefits of binational exchanges of resources and expertise ^16^.

Herein, we present our three-year experience with the international tumor board elucidating the substantial advantages of integrating global expertise into the multidisciplinary management of CNS tumors. Our international tumor board represents a unique and novel initiative. With 39 institutions from 25 countries participating, our program is the largest known international pediatric neuro-oncology tumor board. Our monthly Zoom meetings, which included participants from diverse international institutions, provided a platform for discussing complex pediatric neuro-oncology cases. One of the key outcomes of this initiative has been the enhancement of decision-making, particularly in challenging and rare cases, by incorporating diverse perspectives from experts across multiple countries. This forum has served as a comprehensive platform for the analysis of clinical, pathological, molecular, and radiological data, thereby enhancing diagnostic accuracy and enabling the formulation of more individualized treatment strategies.

While our outcomes share similarities with those of other twinning programs, our inclusion of NF cases is unique. The observed upward trend in NF-specific cases possibly reflect the effectiveness of our outreach and educational initiatives, which likely have encouraged more physicians to seek advice regarding diagnostic evaluations and treatment. Additionally, there has been a significant increase in participant engagement across our international network. The growing number of healthcare professionals participating in our tumor board discussions highlights a strong commitment to collaborative learning and improved patient care. This increase in participation is critical, as it facilitates the exchange of diverse insights and expertise, thereby strengthening our collective ability to discuss cases effectively. The correlation between the rising numbers of participants and cases emphasizes the importance of our ongoing efforts to build a robust, interconnected community, dedicated to advancing the care and management of pediatric neuro-oncology globally.

Furthermore, the collaborations with the NIH enhanced our capacity to conduct complex diagnostic workups when necessary. Real-time access to second opinions and the ability to validate findings against global standards were critical in optimizing patient care. This model exemplifies the potential of international tumor boards to bridge knowledge gaps and enhance clinical outcomes in oncology.

The sustainability of these initiatives relies on the commitment of both participating institutions, the development of strong interpersonal relationships, and the motivation of the collaborating teams ^12,13,26^. Insights from the cross-sectional survey have been instrumental in identifying ways to enhance program sustainability. Key factors influencing regular attendance included effective engagement with experienced leadership and the relevance of the case discussions, which enhanced knowledge and increased the willingness to participate. Consequently, more than 60% of participants attended over five sessions in three years, while an additional third attended between three and five sessions. The barriers to attendance mirrored those reported in prior studies. Aby Arja et al. reported that workload, teleconference timing, and internet connectivity were common obstacles ^13^. Similarly, more than two-thirds of participants cited time constraints due to professional or personal commitments as the main barrier, while language was not a significant issue. These findings offer valuable insights for enhancing program sustainability, promoting international collaboration, and advancing global pediatric neuro-oncology care.

Our report has several limitations, including its retrospective nature and the lack of documented patient outcomes. Additionally, we did not monitor the adherence to the tumor board’s recommendations across all cases or whether they led to improvement in clinical outcomes. We acknowledge that the true impact of these recommendations is contingent upon their implementation, which was not consistently monitored across all cases discussed. Furthermore, due to the lack of baseline data on patients with CNS tumors prior to the initiation of this program, we are unable to compare outcomes or accurately assess the program’s impact on survival rates.

The limitations of our tumor board also mirror those seen in other international collaboration models. Variability in participation, technological infrastructure challenges, and cultural differences are all factors that can impede the smooth functioning of such initiatives ^13,22,27^. In our meetings, participation exhibited variability, fluctuating from month to month. While heightened engagement was observed during the NF-tumor boards, overall participation attained consistency by the third year of the tumor board. We also observed a gradual increment in the number of cases discussed during the sessions, mirroring the rise in the participating institutions over successive years. As of December 2024, our tumor board includes 59 institutions from 30 countries.

## Conclusions

This collaborative model demonstrates the potential of international tumor boards to bridge knowledge gaps and improve clinical outcomes in oncology. The virtual platform offers binational exchange of resources and expertise, dissemination of knowledge, and collaborative research virtually, without the need to travel. We leverage this platform to bring a patient-centered approach to cancer treatment, considering the appropriate level of resources available within their home countries.

## Data Availability

All data supporting the findings of this study are fully included within the manuscript.
